# Metabolomics analysis of three *Artemisia* species in the Tibet autonomous region of China

**DOI:** 10.1186/s12870-022-03513-z

**Published:** 2022-03-15

**Authors:** Xinyu Liu, Jinglong Wang, Enxia Huang, Bo Li, Shuhang Zhang, Weina Wang, Ziyu Guo, Kexin Wu, Yunhao Zhang, Baoyu Zhao, Hao Lu

**Affiliations:** 1 College of Veterinary Medicine, Northwest A&F University, Yangling, 712100 Shaanxi China; 2 Tibet Academy of Agricultural and Animal Husbandry Sciences/State Key Laboratory of Barley and Yak Germplasm Resources and Genetic Improvement, Lhasa, 850002 Tibet China

**Keywords:** *Artemisia sieversiana*, *Artemisia wellbyi*, *Artemisia annua*, Non-targeted metabolomics, LC–MS/MS, Tibet

## Abstract

**Background:**

The *Artemisia* species are widely distributed around the world, and have found important usage in traditional medicinal practice. This study was designed to investigate the metabolites of Tibetan *Artemisia* species and understand the metabolic pathways.

**Methods:**

The metabolites from three *Artemisia* species in Tibet, were analyzed using LC–MS/MS. The differential metabolites were classified and analyzed by principal component analysis (PCA), partial least squares analysis and hierarchical clustering. KEGG Pathway enrichment analysis was used to identify the key metabolic pathways involved in the differential metabolites of three *Artemisia* species.

**Result:**

The metabolites of three *Artemisia* species were analyzed. Under the positive ion mode in LC–MS/MS, 262 distinct metabolites were differentially detected from *Artemisia sieversiana* and *Artemisia annua*, 312 differential metabolites were detected from *Artemisia wellbyi* and *Artemisia sieversiana*, 306 differential metabolites were screened from *Artemisia wellbyi* and *Artemisia annua*. With the negative ion mode, 106 differential metabolites were identified from *Artemisia sieversiana* and *Artemisia annua*, 131 differential metabolites were identified from *Artemisia wellbyi* and *Artemisia sieversiana*,133 differential metabolites were differentially detected from *Artemisia wellbyi* and *Artemisia annua.* The selected differential metabolites were mainly organic acids and their derivatives, ketones, phenols, alcohols and coumarins. Among these natural compounds, artemisinin, has the highest relative content in *Artemisia annua*.

**Conclusions:**

This is the first reported attempt to comparatively determine the types of the metabolites of the three widely distributed *Artemisia* species in Tibet. The information should help medicinal research and facilitate comprehensive development and utilization of *Artemisia* species in Tibet.

**Supplementary Information:**

The online version contains supplementary material available at 10.1186/s12870-022-03513-z.

## Background

*Artemisia* is a large genus of *Anthemideae* in the *Compositae* family. There are about 350 species in the world. The members of *Artemisia* are widely distributed in the temperate, frigid and subtropical regions of the northern hemisphere, with a few species distributed in the southern hemisphere [1]. It is well adapted in various environments and can survive in high altitude and extremely arid areas. *Artemisia* plants are mostly herbs, only a few are bushes or small shrubs, and most of them can be used as medicine and food for human consumption as well as animal feed [2, 3]. There are 186 species and 44 varieties of *Artemisia* plants in China, which are distributed throughout the country and widely used [4] in traditional Chinese medicinal practice utilizing their properties of antibacterial, anti-inflammatory, and coagulant activity [5]. In addition, there are more than 30 *Artemisia* plants distributed in grassland and desert areas. They are highly resistant to the adverse conditions and have potential ecological and economic value [6, 7]. They are important livestock feed, windbreak and sand-stabilizing plants in pastoral areas [8, 9].

*Artemisia* plant extracts contain polysaccharides, essential oils, organic acids, terpenes, flavonoids, with many of these components possessing the anti-inflammatory, immune-regulating, anti-tumor, anti-bacterial and anti-coagulant effects [10, 11]. Artemisinin drugs extracted from this genus of *Artemisia annua* have been demonstrated to be the highly effective anti-malarial therapeutics. The anti-cholera drug "*Artemisia* wormwood" for liver and gallbladder diseases also belong to this genus.

Presently, the types of metabolites of *Artemisia* plants and the differences in metabolites among these plants are not clear. In this study, we selected three *Artemisia* plants for metabolomics analysis using LC-MC/MS methodology to determine the metabolites of these *Artemisia* plants and analyze the differences in metabolites in order to understand the constituents of the 3 species of *Artemisia* in Tibet. This study will provide new evidence for the potential medicinal use of the three Tibetan *Artemisia* species and lay the foundation for further exploration of the active constituents, their metabolic pathways, and pharmacological mechanisms of action.

## Results

### Qualitative analysis of metabolites

The results are shown in the Additional file 1. In the negative ion mode, a total of 220 metabolites were identified from three *Artemisia* species. In the positive ion mode, a total of 535 metabolites were identified from three *Artemisia* species. The results showed that *Artemisia* plants contain polysaccharides, organic acids, flavonoids, terpenes, pigments, coumarin and other chemical components.

### Principal component analysis (PCA)

**Fig. 1:**
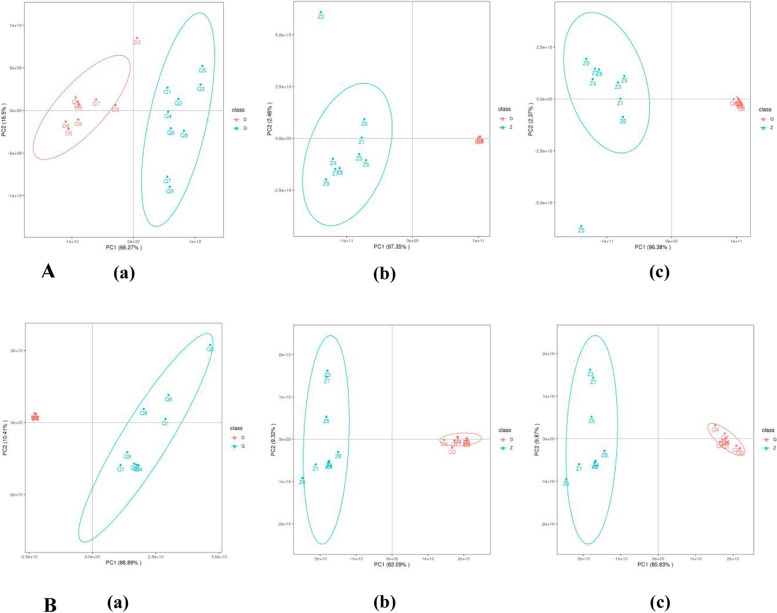
Principal Component Analysis. **A**: Positive ion mode (**a**) D vs Q principal component analysis (PCA) (**b**) Z vs D principal component analysis (PCA) (**c**) Z vs Q principal component analysis (PCA). **B**: Negative ion mode (**a**) D vs Q principal component analysis (PCA) (**b**) Z vs D principal component analysis (PCA) (**c**) Z vs Q principal component analysis (PCA). The horizontal and vertical coordinates PC1 and PC2 in the figure indicate the scores of the first and second ranked principal components respectively, the different coloured scatter points indicate samples from different experimental subgroups, and the ellipses are 95% confidence intervals (95% confidence ellipses cannot be shown when the number of biological replicates is less than 4). (“D” refer to *Artemisia sieversiana*. “Q” refer to *Artemisia annua*. “Z” refer to *Artemisia wellbyi*)

PCA was used to distinguish the overall distribution trend between each two groups of samples (Fig. 1). As shown in Fig. 1A (a) and Fig. 1B (a), the samples of group D are all overlapped, and the correlation is good, while the Q group is mostly separated, and the degree of correlation is not as good as D.. There is no crossover between group D and group Q, which indicate that the difference between the two groups is relatively large, indicating that the metabolites between *Artemisia sieversiana* and *Artemisia annua* have a tendency to separate, and there are differences between groups. As shown in Fig. 1A (b) and Fig. 1B (b), the samples of group D are all overlapped, and the correlation is relatively good. There is no crossover between the D group and the Z group, which shows that the difference between the two groups is relatively large, indicating that the metabolites between *Artemisia wellbyi* and *Artemisia sieversiana* have a tendency to separate, and there are differences between groups. As shown in Fig. 1A (c) and Fig. 1B (c), the samples in group Q are all overlapped, and the correlation is better, while the Z group is mostly separated, and the correlation is not so good. There is no crossover between the Z group and the Q group, which shows that the difference between the two groups is relatively large, indicating that the metabolites between *Artemisia wellbyi* and *Artemisia annua* have a tendency to separate, and there are differences between groups.

### Discriminant analysis of partial least squares (PLS-DA)

**Fig. 2:**
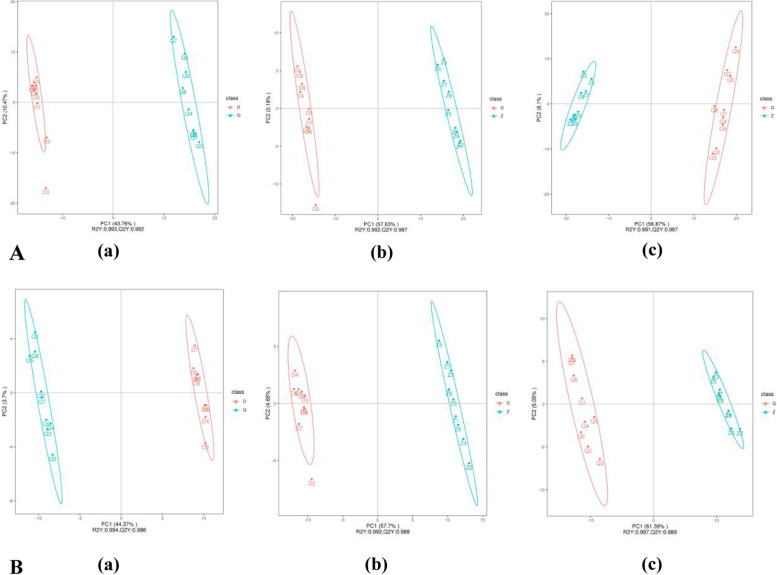
Discriminant Analysis of Partial Least Squares. **A**: Positive ion mode (**a**) D vs Q PLS-DA obtained dispersion plot and sequence verification diagram (**b**) Z vs D PLS-DA obtained dispersion plot and sequence verification diagram (**c**) Z vs Q PLS-DA obtained dispersion Point graph and sorting verification graph. **B**: Negative ion mode (**a**) D vs Q PLS-DA obtained dispersion point diagram and sequence verification diagram (**b**) Z vs D PLS-DA obtained dispersion point diagram and sequence validation diagram (**c**) Z vs Q PLS-DA obtained dispersion point graph and sorting verification graph. Scatter plot of scores, the horizontal coordinate is the score of the sample on the first principal component; the vertical coordinate is the score of the sample on the second principal component; R2Y indicates the explanatory rate of the model, Q2Y is used to evaluate the predictive power of the PLS-DA model, and R2Y is greater than Q2Y indicates a well established model. For the ranking test, the horizontal coordinates represent the correlation between the randomly grouped Y and the original grouped Y, and the vertical coordinates represent the scores of R2 and Q2. (“D” refer to *Artemisia sieversiana*. “Q” refer to *Artemisia annua.* “Z” refer to *Artemisia wellbyi*)

In the group (a) of Fig. 2A and Fig. 2B, the D group and the Q group are clearly separated, which shows that the metabolites between *Artemisia sieversiana* and *Artemisia annua* have a tendency to separate, which can explain the difference between the groups of *Artemisia sieversiana* and *Artemisia annua* is very large. In groups (b) of Fig. 2A and Fig. 2B, there is a clear separation between groups Z and D, demonstrating a trend towards separation of metabolites between *Artemisia wellbyi* and *Artemisia sieversiana* the inter-group differences between *Artemisia wellbyi* and *Artemisia sieversiana* are very large. The clear separation between groups Z and Q in groups (c) of Fig. 2A and Fig. 2B demonstrates the tendency for metabolites to segregate between *Artemisia wellbyi* and *Artemisia annua* and the inter-group differences between *Artemisia wellbyi* and *Artemisia annua* are observable.

### Differential metabolites analysis

The Variable Importance in the Projection (VIP) value of the first principal component of the PLS-DA model was used. The VIP value represents the contribution rate of the metabolite difference in different groups; the difference multiple (Fold Change, FC) represents each metabolism. The ratio of the mean value of the repeated quantitative values of all metabolites in the comparison group; combined with the *p* value of *t*-test to find the differentially expressed metabolites, set the threshold value to VIP > 1.0, the multiple of difference FC > 1.2 or FC < 0.833 and *p-*value < 0.05, and the selected different metabolites are shown in Table 1. The information of the different metabolites selected from the 3 species of *Artemisia* plants is in Additional file 2. Scopoletin was a representative differential metabolite in *Artemisia sieversiana* and Biochanin A was a representative differential metabolite in *Artemisia wellbyi*.

**Table 1:** Statistics of metabolite difference analysis results

Compared Groups	Num. of Total Ident	Num.of Total Sig	Num.of Sig.Up	Num.of Sig.down
D.vs.Q_neg	220	106	38	68
D.vs.Q_pos	535	262	113	149
Z.vs.D_pos	535	312	163	149
Z.vs.D_neg	220	131	69	62
Z.vs.Q_pos	535	306	148	158
Z.vs.Q_neg	220	133	59	74

“D” refer to *Artemisia sieversiana*; “Q” refer to *Artemisia annua*; “Z” refer to *Artemisia wellbyi*

Comparing group D with group Q, in the positive ion mode, a total of 535 metabolites are identified. Among the 535 metabolites, 262 are different. That is, there are 262 differential metabolites between *Artemisia sieversiana* and *Artemisia annua*. Total 149 of differential metabolites are up-regulated among the 262 differentially regulated metabolites. In the negative ion mode, a total of 220 metabolites are identified, and 106 of these 220 metabolites are different. Total 68 of differential metabolites are up-regulated among the 106 differentially regulated metabolites. Comparing group Z with group D, in the positive ion mode, a total of 535 metabolites are identified, and 312 of these 535 metabolites are different. That is, there are 312 differences between *Artemisia wellbyi* and *Artemisia sieversiana* metabolites, of which the total number of differential metabolites that are up-regulated is 163, and the total number of differential metabolites that are down-regulated is 149; in the negative ion mode, a total of 220 metabolites are identified, and 131 of these 220 metabolites are different, that is, there were 131 differential metabolites screened between *Artemisia wellbyi* and *Artemisia sieversiana*. The total number of differential metabolites was 69 up-regulated and 62 were down-regulated.

Comparing group Z with group Q, in the positive ion mode, a total of 535 metabolites are identified. Among these 535 metabolites, 306 are different. That is, there are 306 differential metabolites between *Artemisia wellbyi* and *Artemisia annua*. Among them, the total number of differential metabolites that are up-regulated is 148, and the total number of differential metabolites that are down-regulated is 158; in negative ion mode, a total of 220 metabolites are identified, and 133 of these 220 metabolites are different, namely *Artemisia wellbyi*. A total of 133 differential metabolites were screened from *Artemisia annua*, of which 59 were up-regulated and 74 were down-regulated.

Comparing the difference folds of the different metabolites in the samples of *Artemisia sieversiana* and *Artemisia annua*, as shown in Table 2 are the top 20 differentially expressed metabolic components in the difference fold change. Compared with *Artemisia annua*, clear differences can be seen in *Artemisia sieversiana* regarding the contents of Clotrimazole, Deoxyinosine, Methyleugenol, Scopoletin, Parthenin, Daidzin, Oxymorphone, Gibberellin A3 Nivalenol and several other compounds.

**Table 2:** Significant analysis results of different metabolites (D vs Q)

ID	name	formula	mz	rt	FC
M345T531	Clotrimazole	C_22_H_17_C_l_N_2_	345.116045	531.209	0.000675967
M137T378	2-Pyrocatechuic acid	C_7_H_6_O_4_	137.0230757	377.943	0.001085703
M233T835	Deoxyinosine	C_10_H_12_N_4_O_4_	233.1535352	835.184	0.002761027
M197T515	Vanillylmandelic acid	C_9_H_10_O_5_	197.1168458	515.317	0.003251957
M176T418	Citrulline	C_6_H_13_N_3_O_3_	176.1067365	418.3865	0.004265399
M303T741	4-Coumaroylshikimate	C_16_H_16_O_7_	303.0852229	740.6695	0.004272602
M179T831	Methyleugenol	C_11_H_14_O_2_	179.1063169	830.604	0.005805587
M191T543	Scopoletin	C_10_H_8_O_4_	191.0330383	543.054	0.006373881
M95T359	Dimethyl sulfone	C_2_H_6_O_2_S	95.06065372	359.041	0.007144107
M277T665	Maprotiline	C_20_H_23_N	277.1768268	665.0745	0.007563324
M267T718	Magnolol	C_18_H_18_O_2_	267.1367803	718.218	61.83611633
M165T774	3-Methylxanthine	C_6_H_6_N_4_O_2_	165.0904348	774.339	66.21775279
M185T541	Sebacic acid	C_10_H_18_O_4_	185.1169568	540.671	76.33432461
M245T651	Parthenin	C_15_H_18_O_4_	245.1169082	650.867	84.99636582
M491T560	Malvidin 3-glucoside	C_23_H_25_O_12_	491.1227365	560.099	185.2503201
M417T671	Daidzin	C_21_H_20_O_9_	417.1512452	670.8345	238.9987532
M295T740	Nivalenol	C_15_H_20_O_7_	295.1165284	740.449	248.6474766
M195T534	2-Amino-2-deoxy-D-gluconate	C_6_H_13_NO_6_	195.1740612	534.474	395.244568
M302T583	Oxymorphone	C_17_H_19_NO_4_	302.1378483	582.511	434.4695806
M345T468	Gibberellin A3	C_19_H_22_O_6_	345.1331023	467.938	1064.542676

“D” refer to *Artemisia sieversiana*; “Q” refer to *Artemisia annua*

Differentially present metabolites in the samples of *Artemisia sieversiana* and *Artemisia wellbyi* were compared, and the top 20 differential metabolites in terms of levels of presence are shown in Table 3. Compared to *Artemisia sieversiana*, *Artemisia wellbyi* showed a higher levels of 1-Naphthylamine, Isodehydrocostus lactone, Anastrozole, Pseudoivalin, Etodolac, Prostaglandin I2, Baicalin,Cyanidin 3-O-(6-O-malonyl-beta-D-glucoside), Quercetin, Cyanidin 3-glucoside, Biochanin A, Telmisartan were different in content.

**Table 3:** Significant analysis results of different metabolites (Z vs D)

ID	name	formula	mz	rt	FC
M195T534	2-Amino-2-deoxy-D-gluconate	C_6_H_13_NO_6_	195.1740612	534.474	0.002438344
M143T420	1-Naphthylamine	C_10_H_9_N	143.0692372	420.013	0.003517768
M185T541	Sebacic acid	C_10_H_18_O_4_	185.1169568	540.671	0.004686047
M231T828	Isodehydrocostus lactone	C_15_H_18_O_2_	231.1359405	827.521	0.005209893
M290T357	Argininosuccinic acid	C_10_H_18_N_4_O_6_	290.1230746	357.2365	0.0080221
M232T600_2	Butyryl-L-carnitine	C_11_H_21_NO_4_	232.1537351	599.751	0.009387972
M294T416	Anastrozole	C_17_H_19_N_5_	294.1685475	415.5425	0.011303827
M248T604	Pseudoivalin	C_15_H_20_O_3_	248.1352512	604.322	0.011939235
M288T604	Etodolac	C_17_H_21_NO_3_	288.1586762	604.407	0.015893255
M352T647	Prostaglandin I2	C_20_H_32_O_5_	352.2475222	646.51	0.017394963
M461T444	Luteolin 7-O-glucuronide	C_21_H_18_O_12_	461.0705682	444.0825	619.7752218
M299T771	2-Methoxyestrone	C_19_H_24_O_3_	299.1644825	771.377	707.7741889
M445T468	Baicalin	C_21_H_18_O_11_	445.0745556	467.977	713.5478701
M535T446	Cyanidin 3-O-(6-O-malonyl-beta-D-glucoside)	C_24_H_23_O_14_	535.1065109	445.915	720.6877699
M283T776	Quercetin	C_15_H_10_O_7_	283.0604968	776.127	738.6638856
M449T412	Cyanidin 3-glucoside	C_21_H_21_O_11_	449.1064872	411.589	1281.839102
M285T780	Biochanin A	C_16_H_12_O_5_	285.075064	779.795	1342.352863
M495T661	Telmisartan	C_33_H_30_N_4_O_2_	495.2225081	660.986	1662.944147
M551T520	Quercetin 3-(6-malonyl-glucoside)	C_24_H_22_O_15_	551.1023569	520.204	7822.948434
M301T772	Sphinganine	C_18_H_39_NO_2_	301.1701446	771.638	7864.735938

“Z” refer to *Artemisia wellbyi*; “D” refer to *Artemisia sieversiana*

The different metabolites in the samples of *Artemisia wellbyi* and *Artemisia annua* were compared. Table 4 shows the top 20 differentially expressed metabolic components with differences in fold change. Compared with *Artemisia annua*, *Artemisia wellbyi* is more Clotrimazole, 2-Pyrocatechuic acid, Fenfluramine, Deoxyinosine, 6-Tuliposide A, Chlorpheniramine, Quadrone, Tectorigenin, Biochanin A, Quercetin 3-(6-malonyl-glucoside), Cyanidin 3-O-(6-O-malonyl-beta-D-glucoside.

**Table 4:** Results of significant analysis of differential metabolites (Z vs Q)

ID	name	formula	mz	rt	FC
M345T531	Clotrimazole	C_22_H_17_C_l_N_2_	345.116045	531.209	0.001026279
M137T378	2-Pyrocatechuic acid	C_7_H_6_O_4_	137.0230757	377.943	0.001986427
M113T365	2-Heptanone	C_7_H_14_O	113.0951658	365.093	0.00243751
M145T383	4-Guanidinobutanoic acid	C_5_H_11_N_3_O_2_	145.0844448	383.315	0.00275377
M232T557	Fenfluramine	C_12_H_16_F_3_N	232.1328328	557.387	0.004482499
M176T418	Citrulline	C_6_H_13_N_3_O_3_	176.1067365	418.3865	0.004884859
M233T835	Deoxyinosine	C_10_H_12_N_4_O_4_	233.1535352	835.184	0.005626588
M278T550	6-Tuliposide A	C_11_H_18_O_8_	278.1051472	549.635	0.00571858
M275T700	Chlorpheniramine	C_16_H_19_C_l_N_2_	275.124731	700.117	0.006418824
M249T783	Quadrone	C_15_H_20_O_3_	249.1472448	783.0895	0.007130025
M329T619	Cynaropicrin	C_19_H_22_O_6_	329.1375137	619.393	732.7602317
M267T718	Magnolol	C_18_H_18_O_2_	267.1367803	718.218	751.7935082
M131T835	(E)-3-(4-Hydroxyphenyl)-2-propenal	C_9_H_8_O_2_	131.0486872	835.128	803.6043727
M300T777	Tectorigenin	C_16_H_12_O_6_	300.0571363	777.242	819.6671333
M609T478	Kaempferol 3-O-beta-D-glucosyl-(1- > 2)-beta-D-glucoside	C_27_H_30_O_16_	609.1448457	478.401	1087.629466
M495T661	Telmisartan	C_33_H_30_N_4_O_2_	495.2225081	660.986	1314.763757
M449T412	Cyanidin 3-glucoside	C_21_H_21_O_11_	449.1064872	411.589	1567.046194
M285T780	Biochanin A	C_16_H_12_O_5_	285.075064	779.795	1598.318706
M551T520	Quercetin 3-(6-malonyl-glucoside)	C_24_H_22_O_15_	551.1023569	520.204	1771.05996
M535T446	Cyanidin 3-O-(6-O-malonyl-beta-D-glucoside)	C_24_H_23_O_14_	535.1065109	445.915	2968.958277

“Z” refer to *Artemisia wellbyi*; “Q” refer to *Artemisia annua*

### Volcano map of differential metabolites

**Fig. 3:**
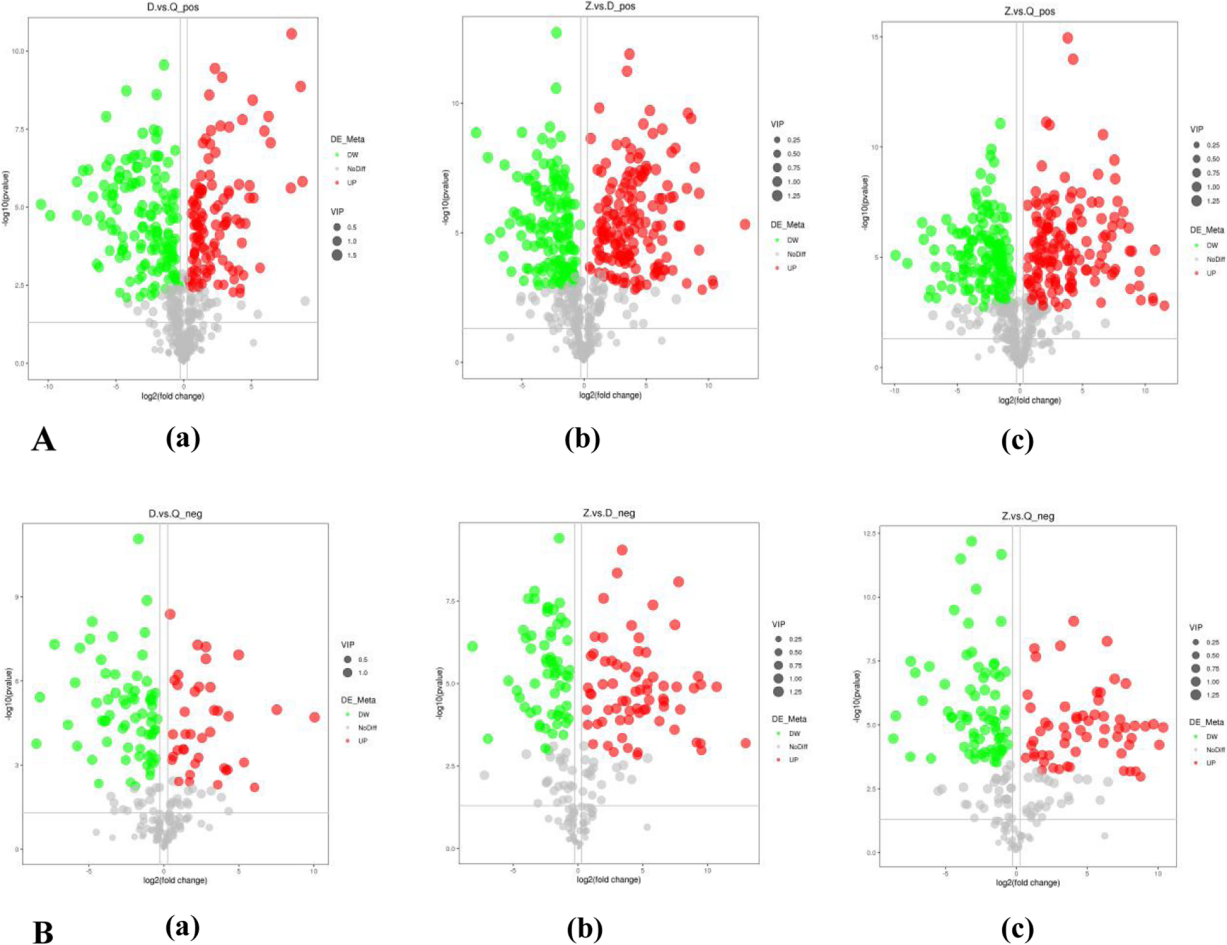
Differential Metabolite Volcano Map. **A**: Positive ion mode (**a**) D vs Q differential metabolite volcano (**b**) Z vs D differential metabolite volcano (**c**) Z vs Q differential metabolite volcano diagram. B: Negative ion mode (**a**) D vs Q differential metabolite volcano diagram (**b**) Z vs D differential metabolite volcano diagram (**c**) Z vs Q differential metabolite volcano diagram. Negative ion mode (**a**) D vs Q differential metabolite volcano diagram (**b**) Z vs D differential metabolite volcano diagram (**c**) Z vs Q differential metabolite volcano diagram. (“D” refer to *Artemisia sieversiana*. “Q” refer to *Artemisia annua.* “Z” refer to *Artemisia wellbyi*)

The volcano chart can visually display the overall distribution of different metabolites, and the results are shown in Fig. 3. Figure 3A and 3B visually show the significantly different metabolites between the three *Artemisia* plants. The overall and visual display of the specific metabolites of each group and their differences can be used as a functional analysis of metabolic pathways. As shown in the Fig. 3, red is up-regulated, green is down-regulated, and gray is not occurring, that is, the metabolites is no difference.

### Cluster analysis of differential metabolites

**Fig. 4:**
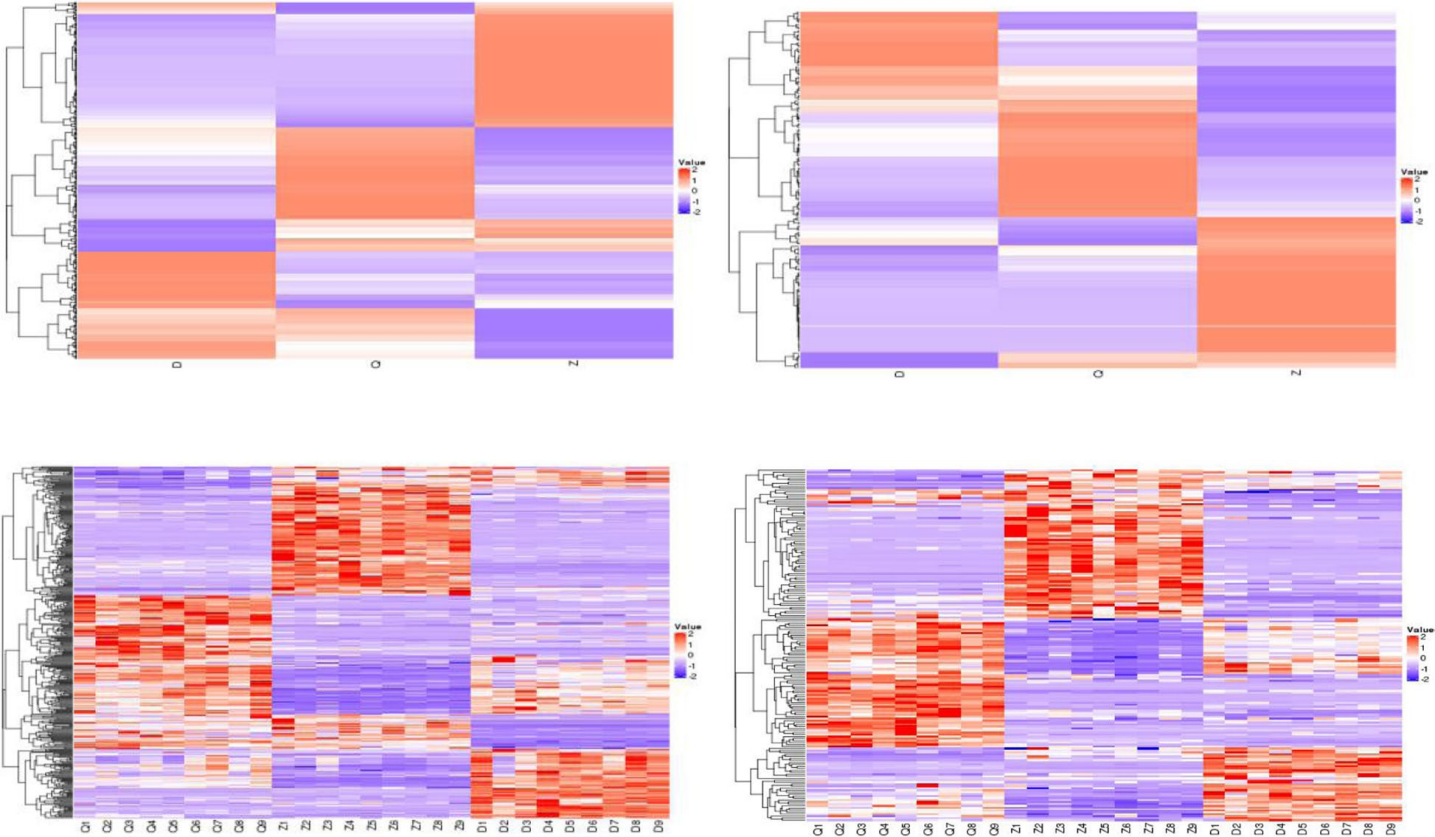
Cluster Analysis of Differential Metabolites. Clustering heat map of total differential metabolites (the upper frame is the grouped heat map, the lower frame is the sample heat map; each frame is the first picture is the positive ion mode, the second picture is the negative ion mode). The vertical direction is the clustering of samples, and the horizontal direction is the clustering of metabolites. The shorter the cluster branches, the higher the similarity. The relationship between the clustering of metabolite content between groups and samples can be seen through horizontal comparison. (“D” refer to *Artemisia sieversiana*. “Q” refer to *Artemisia annua*. “Z” refer to *Artemisia wellbyi*)

A hierarchical clustering analysis is performed on all the difference metabolites between the obtained comparison pairs, and the relative quantitative values of the difference metabolites are normalized and converted and clustered. As shown in Fig. 4.

Different colored areas in the figure represent differently clustered groups.Metabolites with similar expression patterns in the same group will be clustered together suggesting similar or identical biological processes. It can be seen intuitively from the positive ion pattern that the upper part of the Z group is red, and the upper part of the D and Q groups are blue, indicating that there are many different metabolites in the *Artemisia wellbyi* group that are highly expressed, while the expression levels in *Artemisia annua* and *Artemisia sieversiana* group are relatively low.

### Wayne analysis of different metabolites

**Fig. 5:**
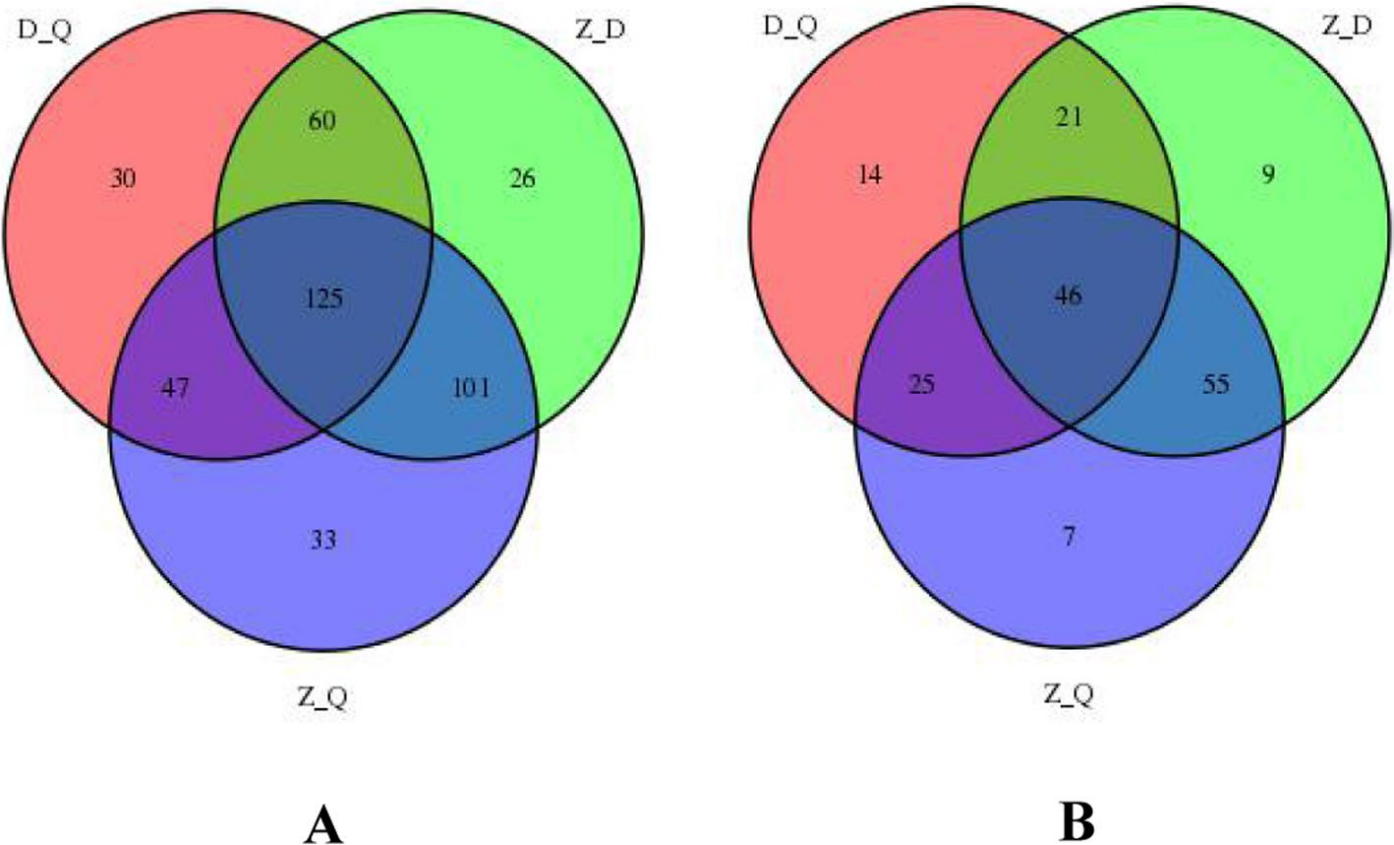
Wayne Analysis of Different Metabolites of Different Species. **A**: Venn diagram of different metabolites of different species in positive ion mode. **B**: Venn diagram of different metabolites of different species in negative ion mode. (“D” refer to *Artemisia sieversiana*. “Q” refer to *Artemisia annua.* “Z” refer to *Artemisia wellbyi*)

In the positive ion mode, the number of different metabolites of different species identified by the multivariate statistical method is 125 (Fig. 5). The number of different metabolites screened by *Artemisia sieversiana* compared with *Artemisia annua* and the different metabolites selected from *Artemisia wellbyi* is 185, the number of different metabolites selected by *Artemisia wellbyi* is the same as that of *Artemisia sieversiana.* The number of different metabolites screened by *Artemisia wellbyi* compared with *Artemisia annua* is 226, the number of different metabolites screened by *Artemisia wellbyi* compared with *Artemisia annua* is the same species. The number is 172.

In the negative ion mode, the number of different metabolites of different species identified by multivariate statistical methods is 46. The number of different metabolites screened by *Artemisia sieversiana* compared with *Artemisia annua* and the different metabolites screened by *Artemisia wellbyi* is 67, the number of different metabolites selected by *Artemisia wellbyi* is the same as that of *Artemisia annua*. The number of different metabolites selected from *Artemisia wellbyi* compared with *Artemisia annua* is 101, the number of different metabolites selected from *Artemisia sieversiana* compared with *Artemisia annua* is the same type. The number is 71.

### KEGG pathway analysis of the metabolites

All the information on the metabolic pathways enriched by the differential metabolites detected in the 3 species of *Artemisia* is listed in Additional file 3. The significance analysis of KEGG can determine the main biological functions performed by the different metabolites. KEGG Pathway enrichment results of different metabolites are shown in Additional file 3. In the positive ion mode, 548 differential metabolites of D vs Q are annotated into metabolic pathways, 741 differential metabolites of Z vs D are annotated into metabolic pathways, and 631 differential metabolites of Z vs Q are annotated into metabolic pathways. In the metabolic pathway, the analysis showed that some metabolites can participate in multiple metabolic pathways, and multiple metabolic pathways are consistent among the comparison groups. In the negative ion mode, D vs Q has 392 differential metabolites annotated into the metabolic pathway, Z vs D has 532 differential metabolites annotated into the metabolic pathway, and Z vs Q has 510 differential metabolites are annotated into the metabolic pathway. The analysis showed that some metabolites can participate in multiple metabolic pathways, and multiple metabolic pathways are consistent among the comparison groups.

### KEGG enrichment bubble chart

**Fig. 6:**
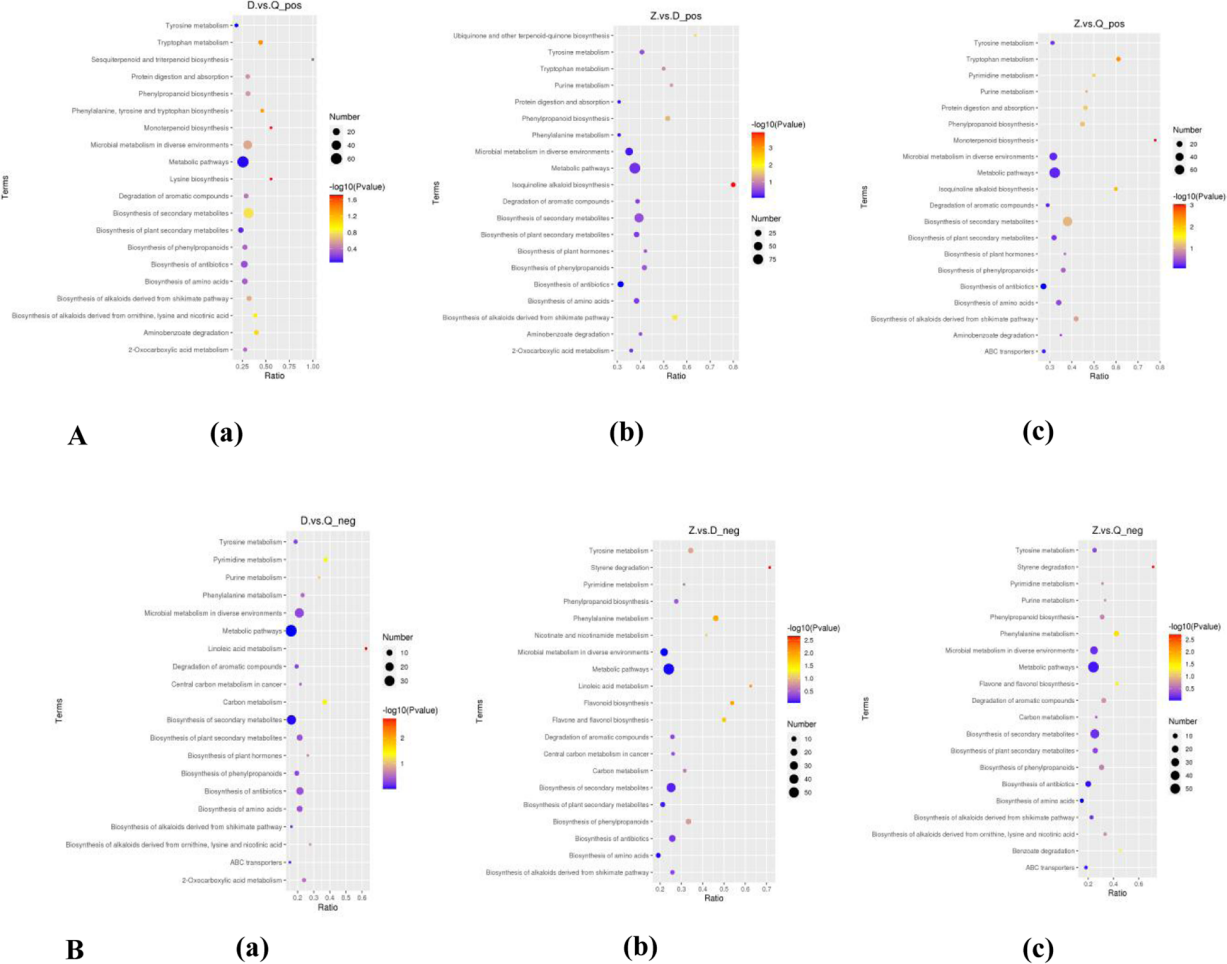
KEGG Enrichment Bubble Chart. **A**: Positive ion mode (**a**) D vs Q KEGG enriched bubble chart (**b**) Z vs D KEGG enriched gas (**c**) Z vs Q KEGG enriched bubble chart. **B**: Negative ion mode (**a**) D vs Q KEGG enriched bubble chart (**b**) Z vs D KEGG enriched gas (**c**) Z vs Q KEGG enriched bubble chart. The abscissa in the figure is x/y (the number of differential metabolites in the corresponding metabolic pathway/the total number of metabolites identified in the pathway). The larger the value, the higher the enrichment of differential metabolites in the pathway. The color of the dot represents the p-value of the hypergeometric test. The smaller the value, the greater the reliability of the test and the more statistically significant. The size of the dot represents the number of different metabolites in the corresponding pathway. The larger the dot, the more differential metabolites in the pathway. (If there is no enrichment result, there is no picture). (“D” refer to *Artemisia sieversiana*. “Q” refer to *Artemisia annua.* “Z” refer to *Artemisia wellbyi*)

The enriched differentially expressed metabolites in KEGG pathway analysis also presented in bubble chart (only the results of top 20) are shown in Fig. 6A, 6B (Fig. 6).

The differential metabolites of *Artemisia sieversiana* and *Artemisia annua* (D vs Q) are enriched in the Linoleic acid metabolism, Monoterpenoid biosynthesis and Lysine biosynthesis pathway. The differential metabolites of *Artemisia wellbyi* and *Artemisia sieversiana* (Z vs D) are enriched in the Styrene degradation and Isoquinoline alkaloid biosynthesis pathway. The differential metabolites of *Artemisia wellbyi* and *Artemisia annua* (Z vs Q) are enriched in the Styrene degradation and Monoterpenoid biosynthesis pathway.

The significant enrichment of these three species of *Artemisia* on these pathways is helpful to understand the metabolic pathways of *Artemisia* plants and their intermediate metabolites, which lays the foundation for their biological research.

### Artemisinin content of three *Artemisia* plants

**Fig. 7:**
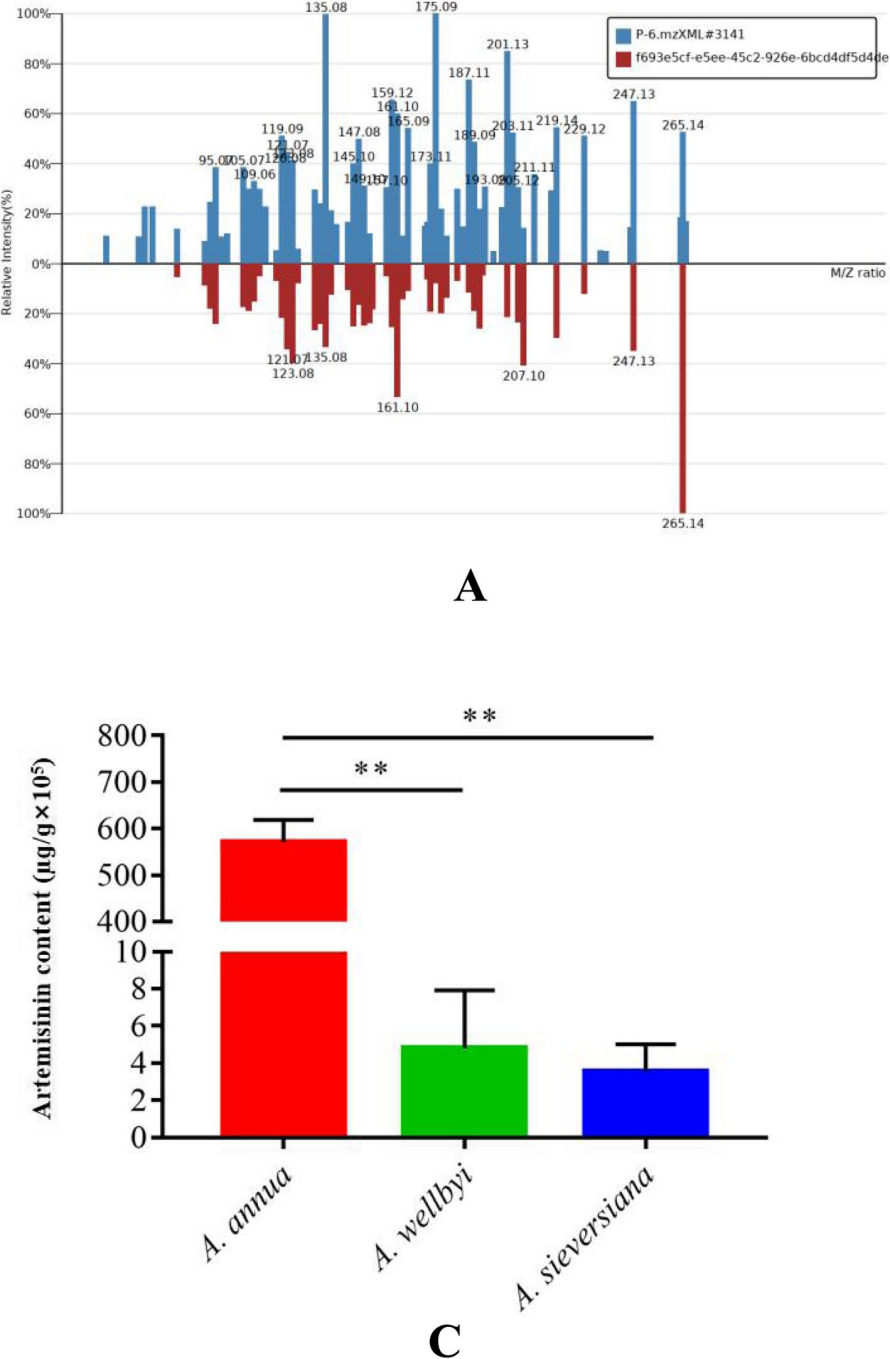
Concentration of Artemisinin in the Plant Material. **A**: The secondary spectrum of artemisinin. **B**: The content of artemisinin in 3 species of *Artemisia*

Based on the detection results of non-targeted metabolomics, we detected artemisinin from three different *Artemisia* plants, and through screening, we found that artemisinin is an important differential metabolite. Figure 7A is the secondary spectrum of artemisinin obtained from three *Artemisia* plants in non-targeted metabolomics.

Having found that artemisinin is an important differential metabolite, we then used high-phase liquid chromatography combined with mass spectrometry to target the artemisinin content in these three *Artemisia* plants. The standard curve was drawn according to the calculated regression equation: Y = 500.74237X + 1551.22512 (R = 0.99980). The concentration of artemisinin in *Artemisia sieversiana* is 3.545 ± 1.202 × 10^5^µɡ/ɡ. The concentration of artemisinin in *Artemisia wellbyi* is 4.799 ± 2.544 × 10^5^µɡ/ɡ. The concentration of artemisinin in *Artemisia annua* is 5.713 ± 0.385 × 10^7^µɡ/ɡ. Compared with *Artemisia annua*, the content of artemisinin in *Artemisia wellbyi* and *Artemisia sieversiana* was lower than that in *Artemisia annua* (Fig. 7B).

## Discussion

In this study, the metabolomics of three representative species of *Artemisia* in blooming stage in Tibet were analyzed by metabolomic technology. The results of metabolite analysis showed that all three *Artemisia* plants contained fatty acids, glycerophospholipids, amino acids, sugars, nucleotides, phenolamines, organic acids, coumarins, catechins, vitamins, indole, and hydroxycinnamic acid. The metabolites of *Artemisia annua* are significantly different*.* Daidzin has a unique effect on breast cancer [12], prostate cancer [13], heart disease [14], cardiovascular disease [15] and other diseases [16]. Scopoletin has been shown to have anti-inflammatory effects [17], anti-tumor effects as well as analgesic effects [18–22]. We study the differential metabolites in *Artemisia wellbyi*. Quercetin has been found to have multiple biological activities, such as antioxidant [21], antiviral [22], and anti-inflammatory effects [23, 24]. Baicalin has significant biological activity. It has antibacterial, diuretic, anti-inflammatory, cholesterol-lowering, anti-thrombosis, relief of asthma, detoxification, and hemostasis [25, 26]. The pharmacological effects of these important metabolites are consistent with those recorded in the published literature [27, 28].

We use LC–MS to target detection of artemisinin content in 3 species of *Artemisia* plants. This study found that artemisinin is present in the three representative *Artemisia* plants, *Artemisia sieversiana*, *Artemisia wellbyi* and *Artemisia annua*, collected from Tibet. *Artemisia annua* contains the highest concentration of artemisinin, with an average value of 57,130 µɡ/ɡ, the second is *Artemisia wellbyi* with an artemisinin content of 479.93 µɡ/ɡ, the last is *Artemisia sieversiana*, its content is 354.47 µɡ/ɡ. Xiang et al. [29] established a quick and easy UPLC-UV method for the detection of artemisinin, and tested the content of artemisinin in *Artemisia annua* from different producing areas, and found that the artemisinin content of *Artemisia annua* from Chongqing City was as high as 10,000.4 µɡ/ɡ. Cheng et al. [30] used UPLC-MS/MS detection to compare the artemisinin content of *Artemisia annua* from different sources, the results found that the origin of *Artemisia annua* with higher artemisinin content was Yunnan province, and the content was 3810.597 µɡ/ɡ, followed by Hainan province, with an average of 3702.952 µɡ/ɡ. By comparison, it is found that the artemisinin content of *Artemisia annua* in Tibet is the highest compared to other provinces, which indicates that as a traditional Tibetan plant of the genus *Artemisia*, *Artemisia annua* has properties of antibacterial, antitumor, antiviral, anti-inflammatory and these pharmacological properties may have important potential medicinal value.

Tibetan medicine is used for anti-inflammatory, visceral bleeding and so on [31]. *Artemisia sieversiana* is also a traditional herbal medicine used by Tibetan and Mongolian medicine. It mainly contains chemical components such as flavonoids, lignins, sesquiterpenes and volatile oils. The medicinal work "Compendium of Materia Medica" mentioned *Artemisia selengensis* and the *Artemisia sphaerocephala* mentioned in "Shen Nong Materia Medica" are all *Artemisia sieversiana* [32, 33]. *Artemisia sieversiana* also has certain medicinal value. According to the records in "The Dictionary of Traditional Chinese Medicine" [34], *Artemisia sieversiana* has a sweet and flat taste, and it mainly treats wind-cold dampness, jaundice, heat dysentery, scabies and malignant sores.

In summary, our results show that Tibetan *Artemisia* plants have broad potential for medicinal value. They are the dominant plants in Tibet's alpine desert grasslands and are also potentially important forage and medicinal plant resources. Moreover, they still play an important role in the ecological protection and economic development of Tibet's grassland. As a plant with both medicinal and edible value, *Artemisia* can also be developed as a functional food at the same time as a high-quality feed for livestock to improve vitality and disease resistance. In future, it is necessary to study the transcriptomics of the genes in these plants to understand their regulation in the synthesis of artemisinin in the three *Artemisia* plants and to transform them by genetic engineering technology to obtain high-yield artemisinin varieties, which can effectively solve the shortage of artemisinin sources.

## Conclusions

This study is based on LC–MS/MS technology to qualitatively determine the differential metabolites of 3 species of *Artemisia* in Tibet. The types of differential metabolites screened out are mainly organic acids and their derivatives, ketones, phenols, alcohols and coumarins. Among them, artemisinin, as a representative differential metabolite, has the highest relative content in *Artemisia annua*. The content is 5.713 ± 0.385 × 10^7^µɡ/ɡ. The key metabolic pathways involved in the different metabolites analyzed by KEGG enrichment are Linoleic acid metabolism, Monoterpenoid biosynthesis and Isoquinoline alkaloid biosynthesis. This study profiled the differential metabolites of the three *Artemisia* plants in Tibet, provided new evidence for their medicinal research, and opened up new ideas for the comprehensive development and utilization of *Artemisia* plants in Tibet.

## Methods

### Plant material

*Artemisia sieversiana*, *Artemisia wellbyi* and *Artemisia annua* were collected in Jinbei, Caina Township, Qushui County, Lhasa City, Tibet Autonomous Region in July 2019. The wild samples in this experiment was permitted by Lhasa Forestry and Grassland Administration. Permission was not necessary for collecting these species, which have not been included in the list of national key protected plants. Te formal identifcation of the plant material was undertaken by Professor Zhaoyang Chang, College of Life Science, Northwest A&F university. The voucher specimens of *Artemisia sieversiana*, *Artemisia wellbyi* and *Artemisia annua* were deposited at Herbarium, Institute of Botany, Chinese Academy of Sciences (voucher number PE01890226,PE01890481,PE01997408). These plants were taken from each sampling site with a size of 10 m × 10 m, and 9 plants were sampled along the diagonal, for a total of 27 samples. All samples were dried, crushed, passed through a 40-mesh sieve (with an aperture of 0.425 mm), put into a paper bag, and stored in a desiccator at room temperature for later use. One g each of 27 samples were wrapped in tin foil, snap frozen in liquid nitrogen for storage, transported in dry ice to Beijing Tiangen Technology Co., Ltd. for analysis.

### Chemical reagents and instruments

Methanol (Merck, Germany), formic acid (ROE, USA), ammonium acetate (Honeywell, USA), and the Mili-Q ultrapure water system comes from Milipore Company (Massachusetts, USA), pipette (Thermo company, USA), freeze dryer, vacuum centrifugal concentrator (Christ company, Germany), centrifuge, mixer (Eppendorf company, Germany), high-speed disperser (IKA company, Germany), 0.22 μm filter membrane (Agilent Company, USA), CPA224S electronic analysis.

### Experimental sample

The 27 plant samples were divided into 3 groups according to 3 different kinds of *Artemisia* plants, the first group "*Artemisia sieversiana*", was indicated by the letter "D"; the second group "*Artemisia annua*", was indicated by the letter "Q"; and the third group "*Artemisia wellbyi*", was marked by the letter "Z". The comparisons between the samples in the group are respectively denoted as D vs Q, Z vs Q, Z vs D, where D vs Q represents the metabolite comparison between "*Artemisia sieversiana*" and "*Artemisia annua*". There were 9 samples in each group, and 3 biological replicate experiments were performed respectively. Quality control samples (QC) were prepared by mixing equal amounts of three *Artemisia* extracts in three replicates and were treated and tested in the same way as the analytical samples, with one QC sample inserted in every 10 analytical samples tested during instrumental testing to investigate the stability and reproducibility of the entire analytical process.

### Metabolite extraction

A 100 mg of liquid nitrogen ground tissue sample was placed in an EP tube, 500 μL of 80% methanol aqueous solution containing 0.1% formic acid was added, vortexed, left to stand in an ice bath for 5 min, and then centrifuged at 15,000 rpm, at 4 °C for 10 min. The supernatant (100µL) was diluted with mass spectrometry grade water to 53% methanol, and placed in a centrifuge tube at 15,000 g, 4 °C for 10 min. The supernatant was collected and injected into LC–MS for analysis. An equal volume of each sample was mixed as QC samples. The blank sample was replaced by aqueous 53% methanol solution containing 0.1% formic acid. The pretreatment process is the same as that of the experimental sample.

### Chromatographic conditions

The chromatography column and conditions are as follows: Chromatographic column: Hyperil Gold column (C18); column temperature: 40 °C; flow rate: 0.2 mL/min; positive mode: mobile phase A: 0.1% formic acid; mobile phase B: methanol; negative mode: mobile phase A: 5 mM ammonium acetate, pH 9.0; mobile phase B: methanol (2) Elution gradient: 98:2 (V/V) at 0 min, 98:2 (V/V) at 1.5 min, 0:100 (V/V) at 12.0 min, 0:100 (V/V) at 14 min, 98:2 (V/V) at 14.1 min, and 17.0 min for 98:2 (V/V).

### Mass spectrometry conditions

Scan range selection was m/z 70–1050 ESI source settings are as follows: Spray Voltage: 3.2 kV; Sheath gas flow rate: 35arb; Aux Gas flow rate: 10arb; Capillary Temp: 320 °C. Polarity: positive; negative; MS/MS secondary scan is data-dependent scans.

### Data processing and analysis

The LC–MS raw data (.raw) files were imported into the CD search software to perform simple screening of retention time, mass-to-charge ratio, and then peak alignment for different samples according to retention time deviations of 0.2 min and massed deviations of 5 ppm were performed. Peak extraction was performed according to the set mass deviation of 5 ppm, signal intensity deviation of 30%, signal-to-noise ratio 3, minimum signal intensity of 100,000 and at the same time the peak area was quantified. The molecular formula of peak and fragment ions was predicted and compared with mzCloud, mzVault and MassList databases. The blank sample was used to remove background ions.

The peaks obtained from all experimental samples were subjected to UV processing and then the data were subjected to PCA analysis (Principal component analysis, PCA) which was used to reduce the dimensionality of metabolite variables through linear combination according to a certain weight, to generate new characteristic variables, and to classify them based on the similarity of the main new variables (principal components) to reflect the overall sample of each group. In order to highlight the differences between the groups and facilitate the subsequent search for different metabolites, the supervised discriminant analysis statistical method was used for partial least square regression PLS-DA, and the PLS-DA model of each comparison group. After sevenfold cross-validation (seven times) cyclic interactive verification, when the number of biological replicates of the sample was n ≤ 3, it is the model evaluation parameters (R2, Q2) obtained by k = 2n). If R2 and Q2 are closer to 1, the model was more stable. To analyze the metabolic patterns of metabolites under different experimental conditions, all the different metabolites between the obtained comparison pairs were clustered into classes for metabolites with the same or similar metabolic patterns for hierarchical clustering analysis. The KEGG Pathway was taken as the unit, hypergeometric test was applied, *p*-value values were calculated. With P*-*value ≤ 0.05 as the threshold, the KEGG term that meets this condition was defined as the KEGG term that was significantly enriched in the differential metabolites. The pathways enriched in differential metabolites were determined comparing with the background of all identified metabolites.

## Supplementary Information

**Additional file 1. **

**Additional file 2. **

**Additional file 3. **

## Data Availability

The datasets used and/or analyzed during the current study are available from the corresponding author on reasonable request.

## Abbreviations

KEGG: Kyoto Encyclopedia of Genes and Genomes
PC1: First principal component
PCA: Principal component analysis
PLS-DA: Discriminant Analysis of Partial Least Squares
LC–MS/MS: Liquid chromatog-tandem mass spectrometry
VIP: Variable importance in projection
